# Separation of Gagua Rise from Great Benham Rise in the West Philippine Basin during the Middle Eocene

**DOI:** 10.1038/s41598-021-01330-2

**Published:** 2021-11-05

**Authors:** Yi-Ching Yeh, Jing-Yi Lin, Shu-Kun Hsu, Ching-Hui Tsai, Ching-Min Chang

**Affiliations:** 1 Department of Earth Sciences, National Central University, Taoyuan, 32001 Taiwan; 2 Center for Environmental Studies, National Central University, Taoyuan, 32001 Taiwan; 3 Institute of Earth Sciences, Academia Sinica, Taipei, 11529 Taiwan

**Keywords:** Ocean sciences, Solid Earth sciences

## Abstract

The West Philippine Basin (WPB) has started opening at ~ 58 Ma and ceased spreading at ~ 33 Ma, developing a fast spreading (~ 44 mm/yr half-spreading rate) magmatic episode between 58 and 41 Ma and the second amagmatic episode between 41 and 33 Ma. The occurrence of the first stage of spreading is closely related to the Oki-Daito mantle plume and related Benham Rise (BR) and Urdaneta Plateau (UP) activity. To the east of the Luzon–Okinawa Fracture Zone (LOFZ), BR was the most active volcanism from 48 to 41 Ma. The geomagnetic ages on both sides of the LOFZ have been determined; however, their causal relationship and evolution in the WPB remain unclear. In this study, we performed integrated analyses of multichannel seismic data and swath bathymetry data for the area to the west of the LOFZ. To the west of the LOFZ, the Gagua Rise (GR), is identified by a high residual free-air gravity anomaly, volcanic seamount chains and an overlapping spreading center. The GR is located at magnetic isochrons C20/C22 (50 to 44 Ma) and shows a thick oceanic crust of at least 12.7 km. We first propose an oceanic plateau named Great Benham Rise (GBR) which includes GR, UP and BR. We infer that the GR was a portion of the GBR since ~ 49 Ma and was separated from the GBR at ~ 41 Ma by the right-lateral LOFZ motion. Later, the relict GBR magmatism only continued in the area to the east of the LOFZ. Overall, the GBR dominates the spreading history of the WPB.

## Introduction

**Figure 1:**
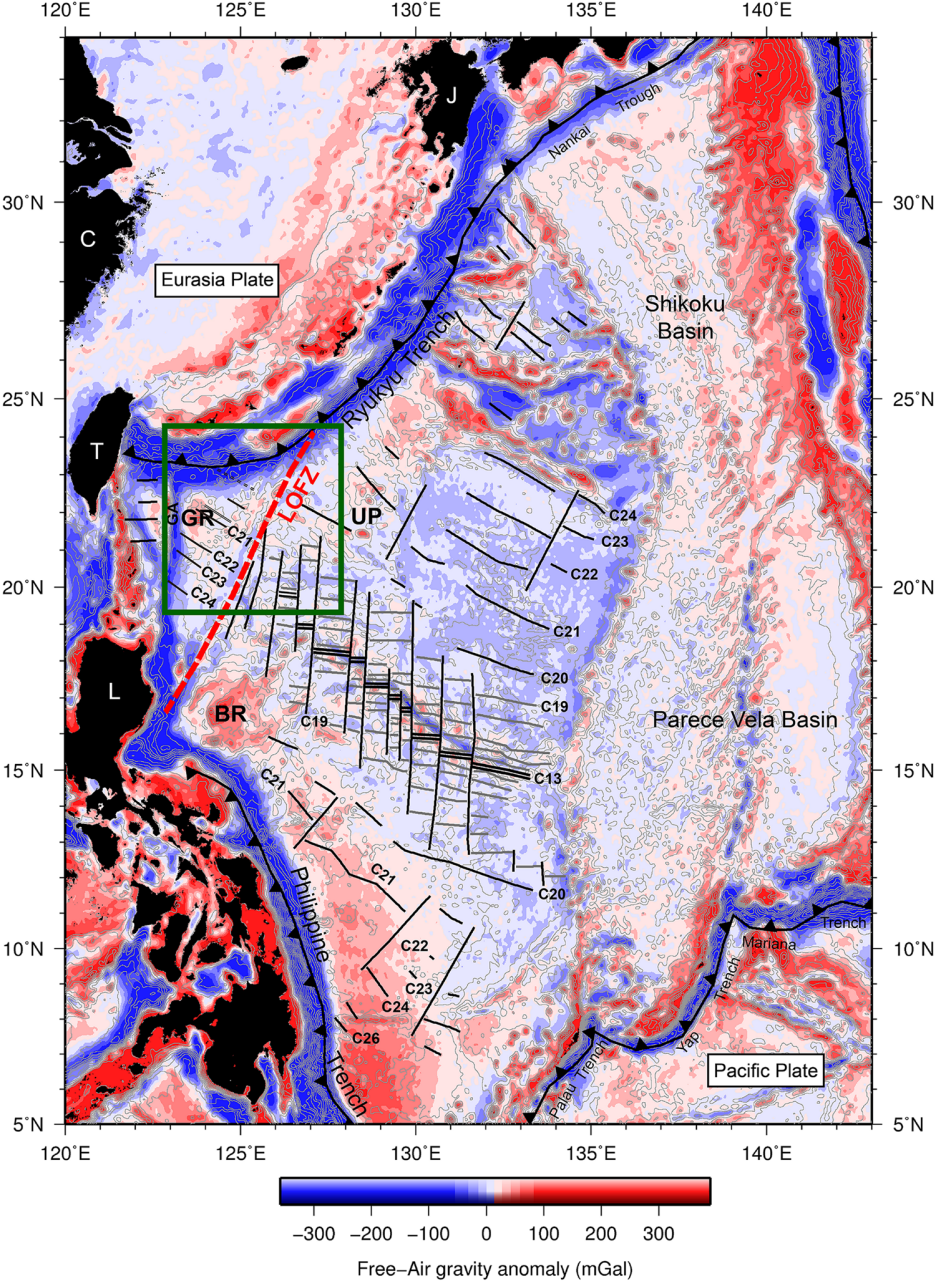
Satellite-altimetry-derived 1 arc min × 1 arc min grid free-air gravity map^18^ of the study area. Identified magnetic anomaly lineations and their age identification^6^ are superimposed on the map. A NE–SW trending spreading occurred between magnetic isochrons C26 and C20 (i.e., 58 Ma and 44 Ma), which then changed to the N–S direction after C20 (i.e., 44 Ma) to the end of the spreading, which occurred at isochron C13 (i.e., 33 Ma). The NE–SW trending spreading episode also appears to the west of the Luzon–Okinawa fracture zones (LOFZ). In the east of the West Philippine Basin (WPB) adjacent to the LOFZ, two relict magmatic topographic rises (Benham Rise in the south and Urdaneta Plateau in the north) sit symmetrical aside the central isochron C13 spreading valley. A relative higher gravity anomaly area (Gagua Rise; GR) is located to the west of the LOFZ similar to the Benham Rise–Urdaneta Plateau pair to the west of the LOFZ. This implies that the Eocene magmatic event affects the west of the LOFZ. GA: Gagua Ridge; BR: Benham Rise; UP: Urdaneta Plateau; GR: Gagua Rise; red bold dashed line: LOFZ; C: Mainland China; T: Taiwan; L: Luzon; J: Japan.

The West Philippine Basin (WPB) is located in the west of the Philippine Sea Plate (PSP). Based on the previous studies on seafloor structures and magnetic anomaly identification, two main opening phases in the entire WPB spreading history was first recognized^1^: The NE–SW direction opening between 58 and 45 Ma and N–S direction opening between 45 and 33 Ma. The entire WPB block was gradually rotated clockwise during the spreading process. The swath bathymetry data and magnetic anomaly data of the relict spreading center region and provided more constraints on the spreading episode^2–4^. They concluded that the seafloor spreading started at ~ 53 Ma and ceased at 33/30 Ma. Before the end of the spreading, a non-magmatic extension^4,5^ created a great depth for the rift valley and was cut obliquely across the former spreading fabrics, which were produced between 30 and 26 Ma (i.e., second episode). In addition, the large supply of basaltic magma in the early stage generated a fast spreading episode (44 mm/yr) at a half-spreading rate and left the quasi-symmetrical relict volcanic topographic rises of the Benham Rise (BR) and the Urdaneta Plateau (UP) (Fig. 1). These two topographic highs correspond to high free-air gravity anomalies (Fig. 1). Nevertheless, a V-shaped seamount chain converged to a local topographic high as a volcanic province, which is suggested to be a relict spreading center at magnetic anomaly reversal C20 (~ 44 Ma)^6^. All these structures are bounded to the west of the Luzon–Okinawa Fracture Zone (LOFZ). To west of the LOFZ, the oldest oceanic crust between Gagua Ridge (GA) and LOFZ was formed at isochron C24 (~ 53 Ma), which was suggested to be a portion of the early staged NE–SW-trending seafloor spreading episode of the West Philippine Basin (WPB)^6^. A ridge jump occurred at magnetic isochrons C20/C21 with a fast-half-spreading rate of 44 mm/yr, which is likely associated with the end of the magmatic spreading episode. However, the spreading history on both sides of the LOFZ, in relation to magmatism particularly during the first stage spreading episode of the WPB is still unclear. In this study, we integrated and analyzed the latest long-offset multichannel seismic data, residual and Bouguer gravity anomaly data derived from altimetry data, and magnetic anomaly data and evaluated seafloor spreading and volcanism interactions from the early to middle epochs of the Eocene in the western corner of the PSP.

### Mantle plume and seafloor spreading interaction

A fast spreading rate and magma supply (e.g., decompression partial melting or hotspot magma) may reduce the strength of the oceanic crust and develop overlapping spreading centers (OSC) rather than transform faults between two spreading ridges^7^. The along-axis seafloor structures of the spreading center consist of a propagating rift and an overlapping spreading center. Once the propagating rift occurs at the oceanic crust, the new oceanic lithosphere pushes the obliquely “doomed” oceanic crust away. Pseudo-faults separate the propagating oceanic lithosphere and form a V-shaped wake, which indicates the propagating direction toward the V-shaped tip^8,9^. The propagating direction also highlights the direction of magma propagation. However, the reason for the initiation of the propagation rift in the oceanic crust is still not clear. The origin of the propagating rift may be related to a hotspot^10^.

The Iceland plume injection developed a V-shaped Reykjanes ridge to the south and Kolbeiney ridge to the north, which is constrained by numerical modeling^11^. In contrast to a propagating rift, the OSC is a non-rigid transform fault that develops at an intermediate to fast spreading rate (i.e., 25–40 mm/yr half-spreading rate), and magma can migrate up or down along the ridge^12^. Previous studies on the East Pacific Rise showed that the upwelling asthenosphere partial melting along the axis may induce the occurrence of OSCs and related seamounts. The magma continues to be injected into the spreading system, which may lead to a local eruption and magma migration away from the source locus along the strike^7,13,14^. This process may lead to the development of OSCs. A previous study applied ^40^Ar/^39^Ar analysis, dating dredged and drilled rock samples in the WPB, suggesting the Oki-Daito mantle plume activity involved seafloor spreading of the WPB since 51 Ma^15^. The geochemistry analysis determined the BR and UP are originated from Oki-Daito mantle plume. In addition to the latest multichannel seismic and gravity modeling study suggests that the volcanism of the BR is likely the origin of the magmatic spreading episode^16^. The most active volcanism stage was between 50 and 41 Ma, and the magma supply continued to 26 Ma. Between 41 and 26 Ma, the UP separated from the BR, which is consistent with the N–S trending second spreading episode^16^. In addition, a V-shaped seamount chain was reported^6^, which is a propagating rift that occurred to the west of the LOFZ. They also found an OSC system adjacent to a V-shaped seamount chain. The age of the OSC system to the west of the LOFZ is similar to that of the six OSC systems to the east of the LOFZ^4,17^. However, previous studies did not mention seafloor spreading dynamics and magmatism to the western LOFZ in relation to the Benham Rise.

## Methods

### Residual and Bouguer gravity anomalies determination

**Figure 2:**
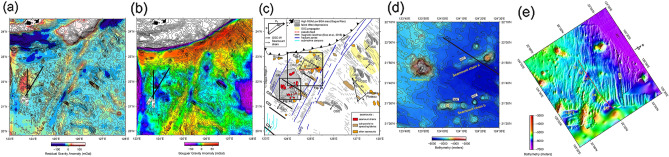
Swath bathymetry map^6^ and its seafloor feature interpretations. (**a**) A 200 m interval swath bathymetric contours with in black superimposed on residual gravity anomaly; bold lines are two long offset multichannel seismic profiles across Gagua Rise; (**b**) Bouguer gravity anomaly map; (**c**) re-interpreted seafloor morphological interpretation map from^6,17,29,30^. (**d**) a close-up figure showing detailed seafloor morphology of V-shaped seamount chains a and b and seamount c. (**e**) a close-up figure showing detailed seafloor morphology of an overlapping spreading center to the west of the LOFZ. A stable N30°E spreading direction is to the west of the LOFZ. In contrast, four-staged counter-clockwise ridge reorientation and jumps (from N42°E to N30°E) are to the east of the LOFZ. However, plenty of seamounts are distributed to the west of the LOFZ as well as an overlapping spreading system and a V-shaped seamount chain. Noted that the seamount chains distributed in between magnetic isochron C22 and C21. The OSC system occurred around magnetic isochron C20. In the southwestern part of the study area, sediments are transported through submarine canyons from the Luzon Island, which blur the seafloor morphology. The spreading direction is relatively stable at N30°E to the west of the LOFZ, which is similar to the area at 126° E, 21° 20′ N to the east of the LOFZ. The double arrows show the orientation of seafloor spreading. IPF: Inner Pseudo Fault; LOFZ: Luzon–Okinawa Fracture Zone; GMFZ: Gagua–Miyako Fracture Zone; LOFZ: Luzon–Okinawa Fracture Zone; OSC: overlapping spreading center; GR: Gagua Rise; RT: Ryukyu Trench; a, b, and c: seamount chains; D1 and D2: residual gravity low depressions; Red dashed line: Inner Pseudo Fault; Black dashed line: paleo spreading center at magnetic isochron C20.

The interaction of volcanism with seafloor spreading between the GA and the LOFZ could be reflected from their composition, i.e. material density. Here, we adopted 1 arc-minute × 1 arc-minute gridded free-air gravity anomaly data derived from altimetry data^18^ from the National Geophysical Data Center (NGDC) database. However, the study area is sandwiched between the Ryukyu Trench (RT) to the north and GA to the west and is approximately 250 km wide in the E–W direction and 400 km wide in the N–S direction. The gravity anomaly is likely contaminated by the long-wavelength effect, known as the subducting plate bending or seamount flexural effect^19^. To eliminate the flexural effect of the West Philippine Sea oceanic lithosphere bending and GA gravity loading, gravity anomaly profiles along a fan-shaped polygon were derived to obtain the long-wavelength gravity anomaly data. Between 121° E and 128° 30′ E, 600 km N–S trending profiles span from the centroid of the axis of the RT (Supplementary Fig. 1). The total profile length is 1200 km (Supplementary Fig. 1). The lateral free-air gravity anomaly profiles derived from the global free-air gravity anomaly data have a grid sampling interval of 10 km (Supplementary Fig. 1). Considering the long-wavelength plate flexural bending gravity effect, we stacked gravity anomaly profiles producing average free-air gravity anomalies (red bold line in Supplementary Fig. 2). The stacked gravity anomaly variations were further assigned to each profile to establish a two-dimensional model grid. The residual gravity anomaly was derived from the difference between the original free-air gravity anomaly and the model gravity anomaly. The same processing procedure strategy was applied to remove the GA flexural effect. The axis of the GA (i.e., highest elevation) is the profiling center where all the E–W trending 1200-km-long free-air anomaly profiles intersect. The profiling interval was also set to 10 km. The stacked gravity anomaly in the GA case is shown in Supplementary Fig. 2. The final residual gravity anomaly is shown in Fig. 2a after removing the long-wavelength component of the gravity anomaly. To better understand crustal accretion in the study area, a Bouguer gravity anomaly map is further determined by replacing water column density (1.03 g/cm^3^) to crust density (2.6 g/cm^3^) ^20^. The result shows in Fig. 2b.

### Magnetic anomaly data and enhanced analytic signal

To better understand magmatic activity and seafloor spreading interactions, we applied an enhanced analytic signal method^21–23^ to identify the magmatic source boundary. Assuming two dimensions in open space, the Hilbert transform of the magnetic anomaly gradient in the depth direction is proportional to that in the *x*-direction^21^. Thus, analytic signal in two dimensions is expressed as follows:

2.3.1 $$ A\left( x \right) = Mx - iMz, \left| {A\left( x \right)} \right| = \sqrt {\left( {\frac{\partial M}{{\partial x}}} \right)^{2} + \left( {\frac{\partial M}{{\partial z}}} \right)^{2} } , $$

where $M\left( x \right) = 2kFc\sin \theta \left[ {\left( {\theta_{1} - \theta_{2} } \right)\cos \emptyset + \sin \emptyset ln\frac{{r_{1} }}{{r_{2} }}} \right]$, $c = 1 - \cos^{2} i\sin^{2} A,\;\emptyset = 2I - \theta - 90^{^\circ }$; *k*: magnetic susceptibility; *F*: magnetic field, *M*: magnetic anomaly; *i*: inclination; *A*: angle between magnetic north and *x* axis. Three-dimensional analytic signal formula was proposed the following^22^:

2.3.2 $$ A_{0} \left( {x,y} \right) = \frac{\partial M}{{\partial x}}\mathop{x}\limits^{\rightharpoonup} + \frac{\partial M}{{\partial y}}\mathop{y}\limits^{\rightharpoonup} + \frac{\partial M}{{\partial z}}\mathop{z}\limits^{\rightharpoonup} . $$

To reduce the interference effect, an enhanced analytical signal method was proposed by taking *n*-th order depth gradient of the magnetic field^23^:

2.3.3 $$ A_{n} \left( {x,y} \right) = \left[ {\frac{\partial }{\partial x}\left( {\frac{{\partial^{n} M}}{{\partial z^{n} }}} \right)\mathop{x}\limits^{\rightharpoonup} } \right] + \left[ {\frac{\partial }{\partial y}\left( {\frac{{\partial^{n} M}}{{\partial z^{n} }}} \right)\mathop{y}\limits^{\rightharpoonup} } \right] + i\left[ {\frac{\partial }{\partial z}\left( {\frac{{\partial^{n} M}}{{\partial z^{n} }}} \right)\mathop{z}\limits^{\rightharpoonup} } \right], $$

where enhanced analytic signal amplitude is defined as follows:

2.3.4 $$ \left| {A_{n} \left( {x,y} \right)} \right| = \sqrt {\frac{\partial }{\partial x}\left( {\frac{{\partial^{n} M}}{{\partial z^{n} }}} \right)^{2} + \frac{\partial }{\partial y}\left( {\frac{{\partial^{n} M}}{{\partial z^{n} }}} \right)^{2} + \frac{\partial }{\partial z}\left( {\frac{{\partial^{n} M}}{{\partial z^{n} }}} \right)^{2} } . $$

Considering that the regional magnetic field follows the Laplace’s equation,

2.3.5 $$ \frac{{\partial^{2} }}{{\partial x^{2} }} + \frac{{\partial^{2} }}{{\partial y^{2} }} + \frac{{\partial^{2} }}{{\partial z^{2} }} = 0, $$

the enhanced analytic signal formula was then modified to

2.3.6 $$ \left( {\frac{{\partial^{n} M_{x} }}{{\partial z^{n} }}} \right)^{2} + \left( {\frac{{\partial^{n} M_{y} }}{{\partial z^{n} }}} \right)^{2} + \left( {\frac{{\partial^{n} M_{z} }}{{\partial z^{n} }}} \right)^{2} = \frac{{\left( {1^{2} \times 2^{2} \times 3^{2} \times \cdots \times n^{2} } \right)\left( {2kFc\sin \theta } \right)^{2} }}{{\left( {d^{2} + x^{2} } \right)^{n + 1} }}. $$

**Figure 3:**
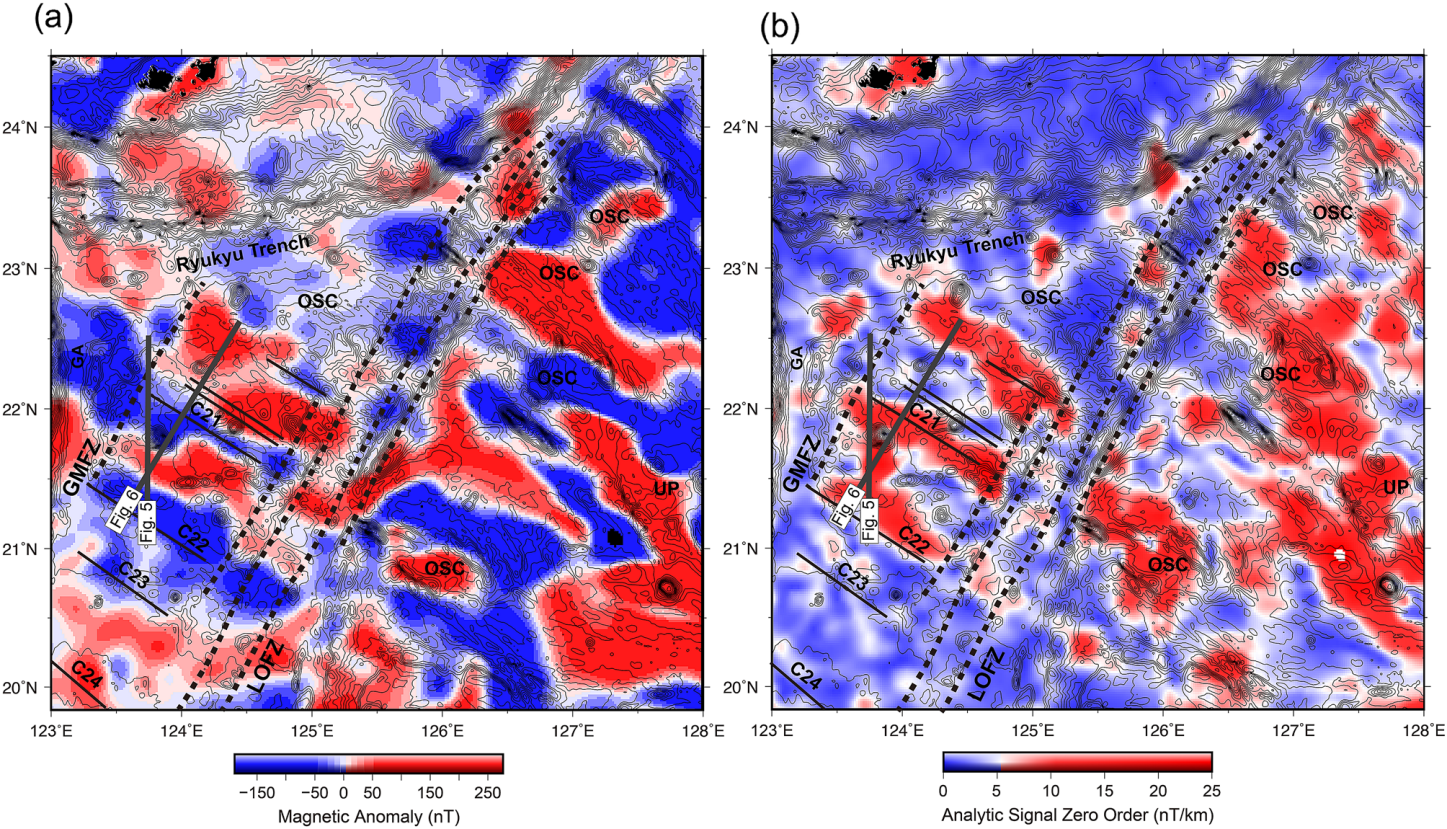
Magnetic anomaly and zero-order enhanced analytic signal map of the study area. (**a**) The latest magnetic anomaly map of the study area^6^; (**b**) Zero-order enhanced analytic signal map of the study area. The gray bold lines are the two long-offset multichannel seismic profiles shown in Figs. 5 and 6. The thin lines represent the magnetic reversal age isochrons. LOFZ: Luzon Okinawa Fracture Zone; GMFZ: Gagua–Miyako Fracture Zone; OSC: overlapping spreading center; UP: Urdenata Plateau.

**Figure 4:**
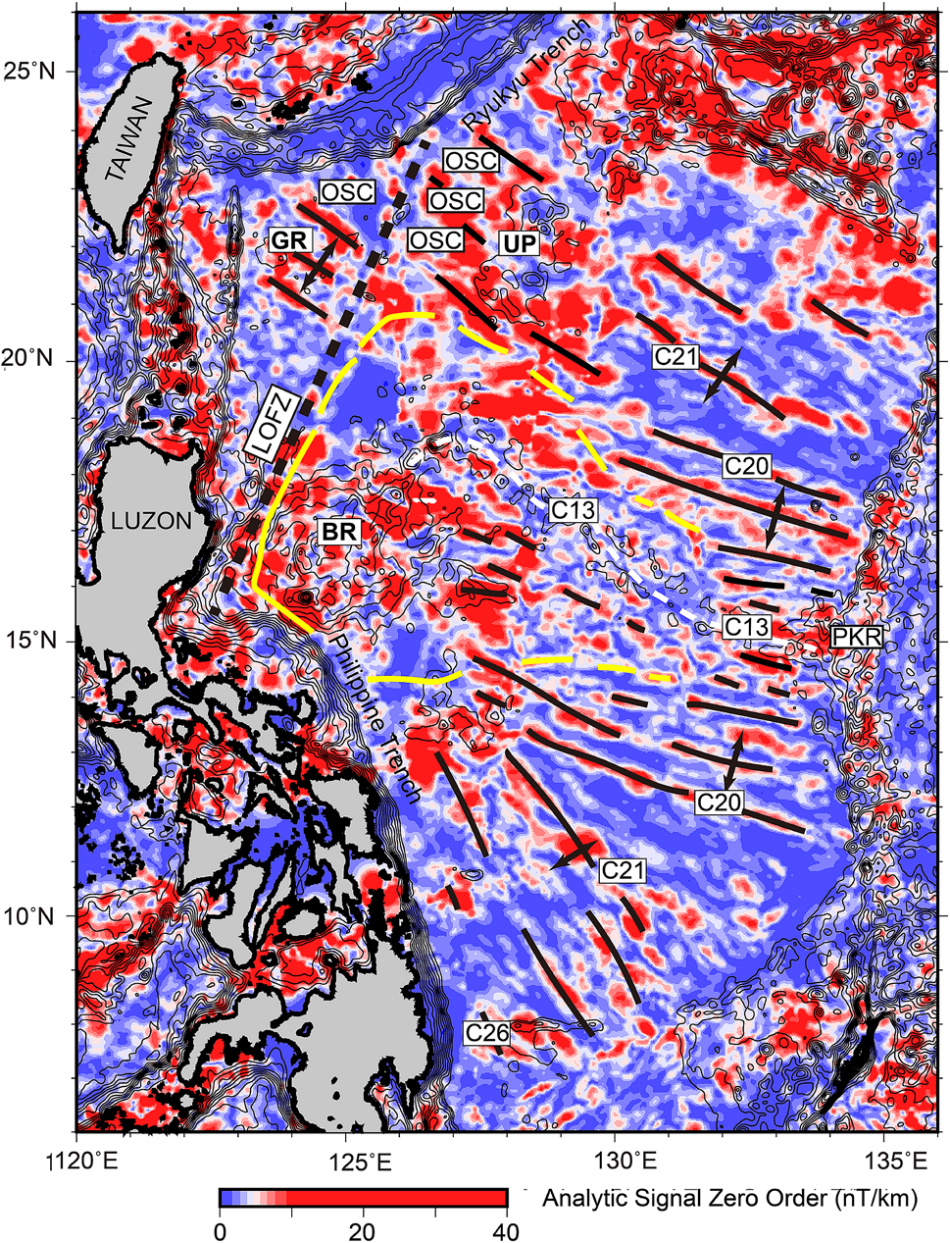
Zero-order magnetic enhanced analytic signal map of the West Philippine Basin. The zero-order enhanced magnetic analytic signal amplitude is higher to the east of the LOFZ than the west. Bathymetric contour interval is 500 m. Bold lines: magnetic isochrons; White dashed line: relict spreading center; Yellow bold line: Benham Rise magma propagation area. BR: Benham Rise; GR: Gagua Rise; UP: Urdaneta Plateau; LOFZ: Luzon–Okinawa Fracture Zone; OSC: overlapping spreading center; PKR: Palau–Kyushu Ridge.

The corresponding zero-order enhanced analytic signal amplitude is defined as follows:

2.3.7 $$ \left| {A_{0} \left( {x,y} \right)} \right|_{max} = \frac{{\left| {2kFc\sin \theta } \right|}}{d}, $$

where *d* is the depth of the magnetic source. The results are presented in Figs. 3b and 4.

### Seismic data processing

The multichannel seismic (MCS) data, collected by the *R/V* Marcus G. Langseth vessel in 2009 under the Taiwan Integrated GEodynamics Research (TAIGER) project, were used in this study. The data acquisition parameters are presented in Supplementary Table 1. The MCS data were processed using the commercial software Landmark Seismic Space ProMAX™ R5000 version, which follows the conventional data processing strategies^24^, including data input, bad/null trace editing, geometry settings, tau-p domain filtering, band-pass filter, F-k filter, time frequency noise rejection, predictive deconvolution, velocity analysis, multiple attenuation, F-X deconvolution, normal moveout correction, stack, and post-stack Kirchhoff time migration. The seismic profile sections in this study were plotted using Seismic Unix (SU) 44R2 free software^25^ and Generic Mapping Tool^26^ version 5. Extraction of the Moho signal is crucial in our analysis, but the Moho reflection is often shaded by low-frequency swell noise and strong reverberation induced by seismic sources before appropriate filtering. This relatively weak coherent reflection is frequently reported in oceanic crust structure studies worldwide^27,28^. Thus, three semblance velocity picking/iteration and constant-velocity stacks derived by semblance velocity picking were executed in this study to enhance the coherence of the Moho reflection.

## Results

### Seafloor morphology and gravity anomaly features

The latest published swath-bathymetric contour map^6^ is presented in Fig. 2a. Figure 2c is a re-interpreted seafloor morphology map which includes morphological interpretations to the west of the LOFZ (this study) and interpretations adopted from previous studies^17,29,30^ to the east of LOFZ. The V-shaped seamount chains a and b (Fig. 2c, d) developed at magnetic isochron C21/C22. The N72° trending seamount chain a is oblique to NW–SE trending seafloor spreading fabrics, converging and developing a round shape seamount at 124° 25′ E, 21° 52.5′ N (Fig. 2d). The N90° trending seamount chain b, is extended eastward to 124° 10′ E and changes direction to be parallel to the seamount chain a, suggesting a typical feature of propagating rift. The V-shaped seamount chain points southeast, suggesting magma propagating southeastward. Figure 2e shows an identified overlapping spreading center (OSC) at magnetic isochron C20^6^ (i.e. black dashed line in Fig. 2e) which developed five stage failed rifted depressions (i.e. D1 to D5 in Fig. 2e). The failed rifted depressions converge toward N30° spreading fabrics, forming a nearly E–W trending inner pseudo fault. The outer pseudo fault cannot be observed in this area which may be due to lack of swath-bathymetric data or subduction beneath Ryukyu trench. Maps of residual gravity anomaly (RGA) and Bouguer gravity anomaly (BGA) of the study area are shown in Fig. 2a, b. Comparison of the altimetry-derived free-air gravity anomaly data (Fig. 1) with the RGA map obtained in this study (Fig. 2a) shows that the RGA magnitude in most areas is reduced to − 50/ + 50 mGal, instead of the extreme value of − 150/ + 150 mGal, at the frontal accretionary prism of the RT. This observation indicates that the flexural effect caused by the subduction of the PSP and GA loading should be removed mostly in the first order. A topographic high adjacent to the Gagua–Miyako Fracture Zone shows a relatively large RGA value (as high as + 50 mGal) and low BGA (from − 20 to − 50 mGal) which suggests existence of crustal accretion (Fig. 2a, b). We named this topographic and RGA high “the Gagua Rise (GR).” To the east of the GR, the area with oceanic spreading fabric has low RGA (approximately − 20 mGal) and high BGA (close to 0 mGal). The seamounts and the LOFZ have a positive RGA value (maximum value reaches ~ 40 mGal) and low BGA value (− 10 mGal to 10 mGal). In addition, the LOFZ reveals high RGA, connecting the highest RGA area (up to 150 mGal) at the frontal accretionary wedge of the Ryukyu subduction zone, which demonstrates the presence of seamounts, fracture zones, and their subduction. This result is consistent with that of a previous study^31^. To the east of the LOFZ, high RGA (10–40 mGal) and low BGA (< − 40 mGal) is associated with seamounts and the UP. Instead of the positive RGA value/ngative BGA value observed for seamounts and oceanic plateaus, seafloor spreading fabrics generally show low RGA and medium BGA (i.e. close to 0 mGal).

### Magnetic features

Figure 3a shows a magnetic anomaly map of the area to the east of the Gagua Ridge. The most dominant structures are the magnetic anomaly stripes, which trend sub-parallel to the NW–SE direction. All the linear features were identified as magnetic isochrons C24–C20 (53–44 Ma)^6^. As high magnetic anomalies are also observed along seamounts, such as the V-shaped seamount chains and the seamounts sub-parallel to spreading fabrics, the magnetic anomaly shows a round shape and irregular magnetic pattern, and the presence of seamounts emplaced on seafloor spreading is related to magnetic strips. To better distinguish magmatic intrusion/extrusion events from seafloor spreading magnetic reversals, we applied the enhanced analytic signal (EAS) method to magnetic anomaly data to enhance the magnetic source boundary. In Figs. 3b and 4, high EAS is associated with magnetic anomaly strips and most seamounts, which are also identified from the RGA, BGA and bathymetry data to the west of the LOFZ (Fig. 2a). To the east of the LOFZ, high EAS coincided with the OSC distribution around the UP, which suggests that the UP could be a magmatic source during the Eocene, in which the OSCs were generated (Fig. 4). According to the EAS distribution to the east of the LOFZ (Fig. 4), three different EAS morphological features can be identified. The first is the NW–SE trending linear feature corresponding to a magmatic spreading episode (magnetic isochron C26 to C20). The orientation of the linear feature changes to the E–W or ESE–WNW directions between magnetic isochrons C20 (~ 44 Ma) and C13 (~ 33 Ma), which reflect an amagmatic spreading episode^4^. The BR shows an irregular high EAS, forming a wedge-shaped EAS boundary toward 131°E (Fig. 4), which indicates that magma propagated southeastward after magnetic isochron C20 (~ 44 Ma). Comparison of the EAS distributions in the study area shows that both sides of the LOFZ show high EAS in general, corresponding to magnetic lineations and seamounts, but the amplitude level to the east of the LOFZ is higher than that to the west, indicating that magmatic activity is more active in the eastern area.

### Seismic data and tectonic feature interpretation

**Figure 5:**
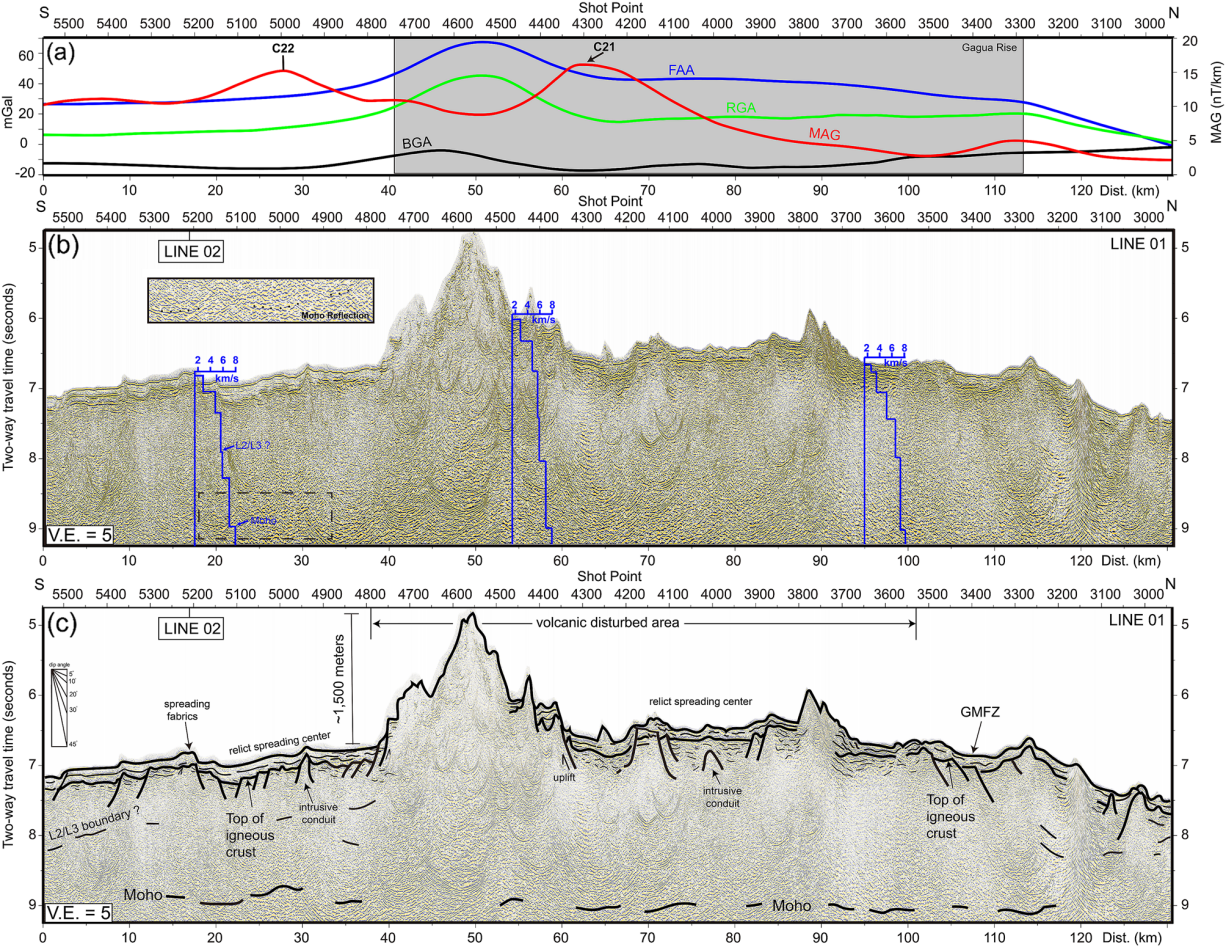
Line 01 long offset multichannel seismic profile section and its interpretation. (**a**) free-air gravity anomaly (FAA), residual free-air anomaly (RGA); Bouguer anomaly (BGA), and zero-order enhanced analytic signal (MAG) along seismic section; (**b**) time-migrated seismic profile section; (**c**) seismic interpretations overlie on the time-migrated seismic profile section. GMFZ: Gagua–Miyako Fracture Zone. Blue step bold lines on (**a**): internal velocity variations from MCS velocity analysis.

**Figure 6:**
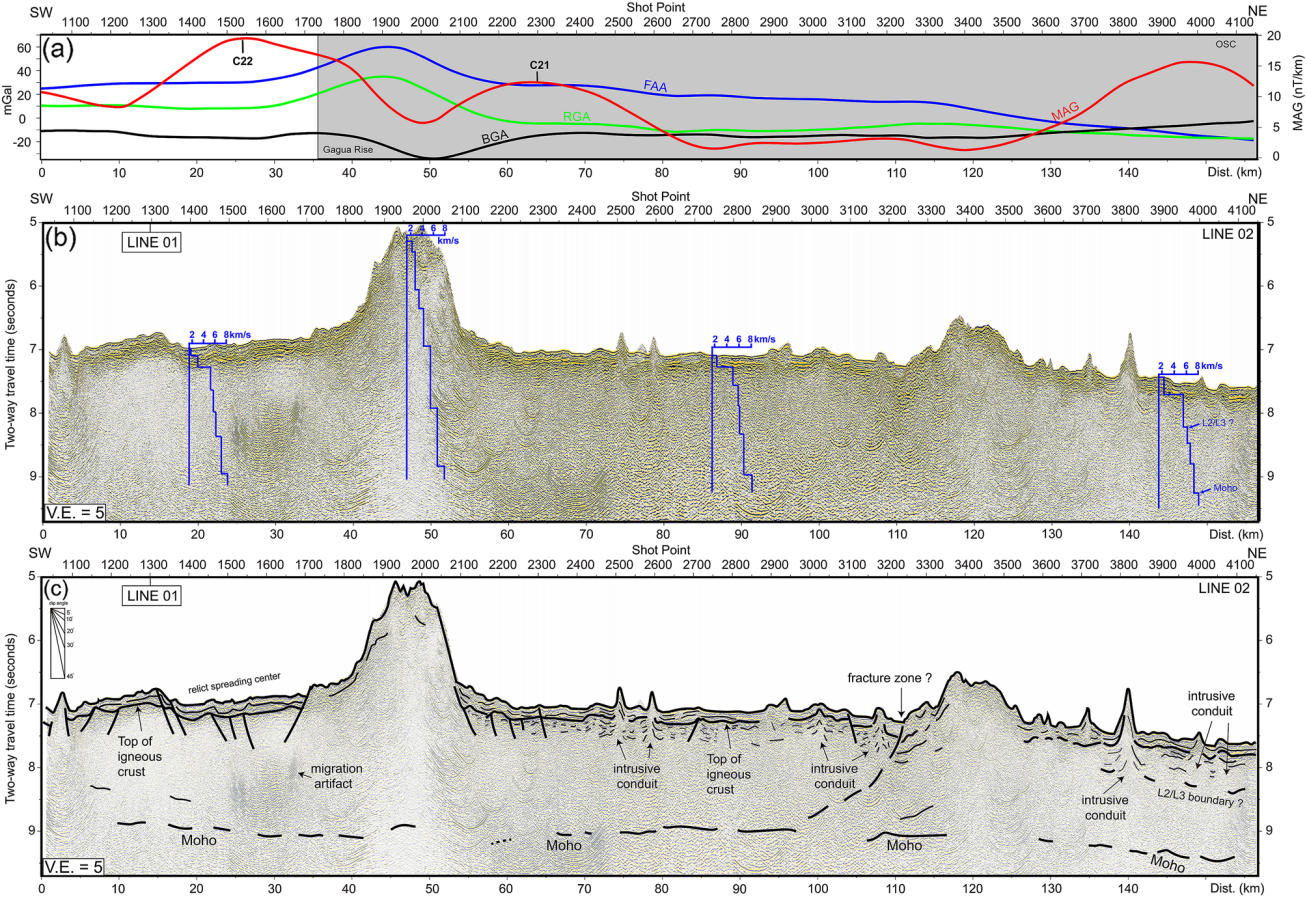
Line 02 long offset multichannel seismic profile section and its interpretation. (**a**) free-air gravity anomaly (FAA), residual free-air anomaly (RGA); Bouguer anomaly (BGA), and zero-order enhanced analytic signal (MAG) along seismic section; (**b**) time-migrated seismic profile section; (**c**) seismic interpretation overlie the time-migrated seismic profile section. Blue step bold lines on (**a**): internal velocity variations from MCS velocity analysis.

To better understand the crustal structures and volcanism interactions with seafloor spreading in the study area, two long-offset MCS profile sections were selected across the high-RGA and low-BGA province (i.e., GR) located to the east of the GA (Figs. 2 and 3). To better trace the Moho reflections and inter crustal reflections (i.e. oceanic L2/L3 interface), seven internal velocity models derived from MCS velocity analyses are superimposed on raw MCS profile sections (Figs. 5 and 6). The velocity variations of the Moho reflection is from 6.8 to 8 km/s and possible oceanic L2/L3 interface is from 4.5–5.2 to 6–6.5 km/s. The MCS profile sections are shown in Figs. 5 and 6, and their interpretations are presented in below two sections.

### Seismic profile section line 01 (Fig. 5)

Line 01 is a N–S trending seismic profile that crosses the high-RGA/low-BGA province (GR) to the south of the RT (Fig. 1). The topography is relatively gentle and smooth in the south of the shot point 4800 (position 0–37 km). Below the seafloor, a weak to blank seismic amplitude sequence (0.5 two-way travel time, T.W.T., of less than 500 m thick) is a sub-parallel seafloor, which is a typical feature of pelagic sediments in deep-sea basins^32,33^. Below the sediment sequences, a semi-continuous and strong amplitude reflection was observed with positive polarity, which was identified as the top of the igneous rock. The basement is cut by 30° normal faults dipping southward at position 0–15 km, which is identified as a seafloor spreading fabric or a rifting shoulder. A symmetrical local depression is located between positions 18 and 27 km. In the north of position 27 km, the basement shoals are elevated up to 4.8 s T.W.T. (to 1500 m elevation). At positions 50–65 km, the sediment strata tilt upward to 6.5 s T.W.T. developing a 0.5 s T.W.T. (375 m) topographic drop accompanied by extrusive volcanism (at position 57 km). The basement relief is sub-parallel to the seafloor morphology and uplifted, causing a high-angle normal fault, which indicates that intrusive or extrusive volcanic activity may occur during onset or post-spreading episodes. At positions 18–38 km, a symmetrical depression corresponds to the magnetic isochron C22, which is likely a relict spreading center. Another symmetrical depression is located between 60 and 85 km, corresponding to a younger magnetic isochron C21. This is consistent with the results of a previous magnetic identification study^6^. At positions 62–120 km, a series of volcanic extrusions cause a hummocky seafloor (position 62–70 km; position 85–95 km). The seafloor and associated basement dip northward around position 115 km close to the RT. The northward dipping normal faults are a consequence of the plate bending effect. Nevertheless, among these profiles, the Moho reflection was identified as the deepest semi-continuous and medium /diffuse amplitude reflector, as shown in the close-up figure in Fig. 5. The Moho reflection depth varies from 9.2 to 8.8 s T.W.T. The average Moho depth is approximately 8.8 s T.W.T. in the south of the seamount and dips northward down to 9 s T.W.T. (position 54 km), indicating oceanic crust thickening under the volcanic disturbance area (position 40 to 105 km). The RGA is positive (from 10 to 40 mGal); BGA is negative (from − 20 to − 5 mGal) between 20 and 110 km, which implies a thick oceanic crust or upper mantle material shoaling. In addition, EAS shows relatively high values at positions 43 and 115 km, marked by the boundary of the volcanic intrusion/extrusion: Gagua Rise (GR).

### Seismic profile section line 02 (Fig. 6)

Line 02 is a NE–SW trending seismic profile, which is perpendicular to the seafloor spreading direction in the WPB (Fig. 1). At position 0–30 km, the sediment sequences are divided into two sections: (1) weak to blank seismic reflection characteristic in the upper section, which are likely of pelagic origin; (2) discontinuous and strong amplitude characteristics in the lower section associated with volcanic extrusion or volcanic breccia deposits. The total thickness of sediment strata is approximately 0.5 s T.W.T. (i.e. less than 500 m). A discontinuous and strong amplitude reflection underneath the sediment strata is identified as the top of the igneous rock. The stepped basement cut by a 30° normal fault hanging southward from 0 to 15 km indicates an oceanic crust spreading fabric. In the north of position 15 km, a symmetrical local depression is observed, which is also found in profile Line 01. Similar to Line 01, this depression corresponds to the C22 magnetic isochron, which is likely a paleo-spreading center. At positions 32 and 55 km, a basement pop-up feature forms a seamount with 1500 m maximum elevation. This amount is the same as that shown in Fig. 5. In the north of position 55 km, the basement is relatively smooth and distributed at 7 s T.W.T., similar to the volcanic undisturbed area (position 0–32 km). Distinct spreading related normal faults can be found at positions 55–65, 82, and 102 km. The upward dragging strong amplitude reflections are adjacent to small-scale seamounts, blanking to chaotic reflection characteristics inside the seamount, indicating intrusive magma conduits at positions 74, 78, 100, 107, 140, 148, and 152 km. In addition, a semi-continuous reflector is about 500 m below the basement, which may be an oceanic crust L2/L3 interface at 135–152 km. The Moho reflection depth varies from 8.8 to 9.5 s T.W.T. amongst the profiles. The RGA is positive (10 to 20 mGal; maximum value 20 mGal at seamount) and BGA is negative (− 34 to − 10 mGal; minimum value − 34 mGal at seamount) to the south of position 50 km. Another local high RGA corresponds to the same value shown in Fig. 5 at positions 110–130 km. A positive-RGA/low-BGA area bounded by two high EAS picks indicates a seamount originating from magma intrusions/extrusions at positions 38 and 65 km. Another EAS high located at position 148 km is associated with an OSC system identified by bathymetry data, suggesting the existence of magma accretion.

## Discussion

### Moho reflections of Gagua Rise (GR) and other plumes—spreading interaction systems

Due to the lack of refraction seismic data, we adopted average oceanic crust P-wave velocity 6.35 km/s (i.e., oceanic crust Layer II: 4.55 km/s; Layer III: 7.1 km/s^34,35^) to estimate the crustal thickness in the study area. The seismic data interpretations in seismic Line 01 show that the Moho depth is located at approximately 2 s T.W.T. (~ 6.35 km) below the acoustic basement in the area where there is less volcanic disturbance. The crust becomes thicker toward the seamount (position 50 km), which is up to 4 s T.W.T. (~ 12.7 km thick). Line 02 seismic profile section shows a thinner crust of 1.8 s T.W.T. (5.7 km) in the less volcanic-disturbed area. Below the seamount (position 50 km in Line 02), crustal thickness is 4 s T.W.T. (12.7 km), similar to that observed along Line 01. Previous studies applied multichannel and refraction seismic methods to illustrate and image Moho depth reflections in spreading/plume interaction areas. The most active spreading/plume interaction system is in Iceland and showed the accretion of large-volume oceanic materials under the oceanic crust during the early stage oceanic crust formation, developing an anomalous 9–13 km thick oceanic crust off eastern Iceland^36^. In the Shasky rise of the Western Pacific Plate, the mantle plume caused the largest magma accretion to develop a 35 km thick oceanic crust^37^. There are many factors that affect the Moho reflection amplitude and continuity. For instance, the Moho reflections show relatively strong amplitudes but discontinuous patterns in the vicinity of the crustal accretion area, which suggests that seafloor complexity (i.e., rough seafloor due to seamounts) may cause strong surface scattering, reducing the seismic source energy^37^. An OBS/MCS joint study in the northeast of the Hawaii Islands showed a diffuse and discontinuous Moho reflection^28^ with survey lines close to the relict spreading center. The relict upper mantle material may cause a Moho reflection blur. In the Galapagos spreading center of the eastern Pacific area, the Galapagos mantle plume interacts with the spreading center, thickens the oceanic crust, and shows discontinuous-/strong-amplitude Moho reflections^38^. The Galapagos hotspot provides magma to the Galapagos spreading center, forming V-shaped seamount chains, which is similar to the observations in our study (Fig. 2). Overall, the existence of a mantle plume or a hotspot can increase seafloor roughness and develop diffuse/discontinuous Moho reflections. In our study area, most Moho reflections show medium amplitudes and discontinuous patterns, which is consistent with the crustal accretion by the mantle plume or the hotspot interacting with the seafloor spreading origin. The BR to the east of the LOFZ, located in the southwestern Philippine Sea Plate, was identified as a local hotspot developing a 15-km-thick oceanic crust during the Early to Middle Eocene^16^. Compared to the BR, the relatively thin crustal thickness (12.7 km) of the GR, which developed in the same period as the BR (i.e., Middle to Early Eocene), is located to the west of the LOFZ. This consistency implies that the magma supply is shorter on GR than on BR during that time.

### Magma propagation rate estimation to the west of the LOFZ

Before estimating magma propagation rate in the study area, age identification needs to be discussed. The study area is sandwiched between GA and LOFZ (Fig. 1). The seafloor fabrics is NW–SE trending, consistent to the magnetic stripe direction (magnetic isochron C26 to C20) to the east of the LOFZ. The determination of the latest magnetic age identification model in the study area^6^ has considered the DSDP site 293 core drilling down to oldest volcanic breccia just above basement, dated as ~ 42 Ma^39^ to the west of the LOFZ which is similar to the age of the generation of UP to the east of the LOFZ^15^. A previous plate reconstruction study in the WPB also suggests our study area is probably a portion of WPB^4^. Although the latest radiometric study based on the dredged samples from GA suggests a Cretaceous relict arc volcanism origin, suggesting GA maybe part of a Cathaysain continental fragment^40^. The Huatung Basin might be an early Cretaceous Ocean domain^40,41^. However, the GA is suggested to be a paelo transform boundary^20^ separating significantly E–W trending Huatung Basin to west from NW to SE trending seafloor fabrics to east. The age should be different on both sides. The geomagnetic age model used in this study is reasonable. Since magnetic age identification still contains uncertainty, future dredge/drilling can further test proposed hypothesis when cruises are available.

Seamounts in our study area can be classified into three types based on their location and orientation. (1) The N30°E trending seamount is sub-parallel to the seafloor spreading fabric and is also adjacent to the fracture zones (Fig. 2c, d). This means that the magma may be injected through the weak zones in the crust (i.e., fracture zones or faults) and then develop seamounts. These seamounts also overlie the pre-dominant fracture zones following the spreading fabrics, which indicates that this volcanic event occurred at the onset or after the cessation of the LOFZ (i.e., the principal of cross-cutting). (2) Two V-shaped significant seamount chains were identified in a previous study^6^ in this area, which are located at 21° 30′ N, 124° 10′ E and 21° 50′ N, 124° 20′ E (seamounts a, b, and c in Fig. 2c, d). Unlike the seamounts observed along the spreading fabrics and fracture zones, these two seamount chains are oblique to the N30°E spreading fabrics in the vicinity of the OSC area. Seamount chain a also developed a V-shaped pattern identical to that of the OSC propagator without disturbing the seafloor spreading features (Fig. 2c, d). There is a slight difference between the seamount chains a and b. Seamount chain a remains oriented at N72°E, but seamount chain b originates at N90°E and then changes to N72°E (Fig. 2c, d). This implies that seamount c had a fast-propagating rate at first and maintained a coherent propagation rate as seamount b (i.e., assumed constant half-spreading rate). (3) The seamount chains found in the Hawaiian Islands^42^ in the Western Pacific originated from a fixed hotspot relative to the overlying oceanic lithosphere. The orientation of the seamount chains in Hawaii marked the plate motion trend or plate re-orientation. However, once the plume source interacts with the active oceanic spreading system, the seamount chain orientation reveals a V-shaped pattern, such as in our study area. The open angle of the V shape is controlled by the spreading rate and magma propagation rate. By measuring the angle between the inner pseudo-fault, propagating ridge θ, and half spreading rate φ by identifying the magnetic anomaly, the propagation rate p of an OSC can be computed; i.e., p = φ⁄tan θ (Fig. 2b)^43^. To estimate the magma propagation rate φ on both sides of the LOFZ, a half-spreading rate of 44 mm/yr at magnetic isochrons C20/C21 (44 Ma/48 Ma) from the latest magnetic lineation age was adopted^6^. θ is approximately 40° for the OSC–W feature in Fig. 2c. Thus, the propagation rate of the OSC–W (p1) is approximately 52.4. In contrast to the OSC–W, the θ value of OSC–E in Fig. 2c is approximately 25°, which indicates that the propagation rate of the OSC–E (p2) value is 94.4 mm/yr. p2 is larger than p1, indicating that the magma source in the east of the LOFZ was larger than that in the west of the LOFZ. This result explains why there are more than six OSCs and ridge jumps to the east of the LOFZ^17^. Furthermore, seamount c that sits at the westernmost end of the V-shaped seamount chain can form a vector headed toward the tip of the chain. This vector can be decomposed into the NE–SW trending spreading rate and NW–SE trending magma propagation rate parallel to the seafloor fabric. Comparison of the results of the OSC–W (black triangle in Fig. 2c) and the OSC–E (gray triangle in Fig. 2c) shows that both the half-spreading rate and propagation rate increased from south to north (close-up in Fig. 2c). The V-shaped seamount chains and GR locate in between magnetic isochron C22/C21(50 Ma/48 Ma) which indicates that the magma activity to the west of the LOFZ likely occurred since ~ 49 Ma, which is very close to the Oki-Daito mantle plume arriving WPB time period^15^.

### Potential kinematic model in the west corner of the West Philippine Basin

The latest magnetic anomaly identification study^6^ suggested that the NW–SE trending oceanic domain between the GA and LOFZ, dated from 54 to 44 Ma (magnetic isochrons C24 to C20), may be a part of the main WPB. There are two possible explanations for the origin of volcanism in the study area: isolated magma source or part of the BR^6^. However, a previous study^6^ did not discuss the interactions between seafloor spreading, volcanism, and plate dynamics. The latest geochemistry analysis on rock samples to the east pf the LOFZ suggested an Oki-Daito mantle plume affecting seafloor spreading of the WPB since 51 Ma^15^ developing BR and UP. In addition, the latest seafloor morphology, multichannel seismic data, and forward gravity modeling data in the BR, located in the east of the LOFZ, suggest that the mantle plume interacted with the seafloor spreading between 50 and 26 Ma^16^. The most active volcanism occurred from 50 to 41 Ma, which may have developed the UP. The UP separated from the BR after 41 Ma (magnetic isochron C19), which began the N–S trending spreading episode until the cessation of the seafloor spreading of the WPB (magnetic isochron C13). To the west of the LOFZ, V-shaped seamount chains and OSC also occurred at magnetic isochrons C20/C22 (44 Ma/50 Ma), which is consistent with the active volcanism period^15,16^. In addition, volcanism is not only limited to the RGA-/EAS-high area but also close to the LOFZ (i.e., seamounts follow spreading fabrics in Fig. 2c) and the RT area (i.e., the unique type seamount in Fig. 2c), which suggests that the area north of 21°N latitude between the GA and LOFZ was affected by volcanism during the seafloor spreading episode magnetic isochron C20 to C22. This is consistent with the results of the previous studies^4,6,15,16^.

**Figure 7:**
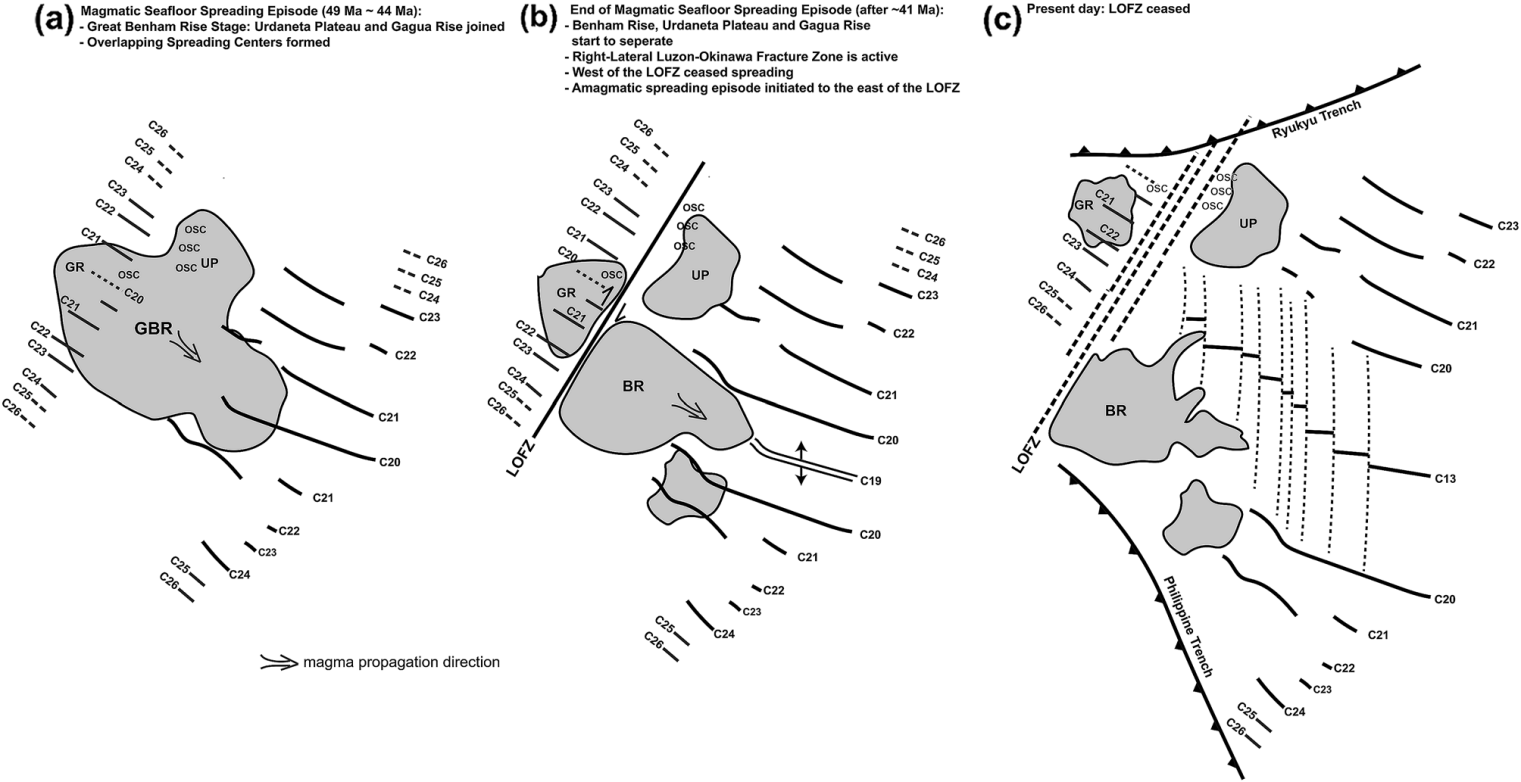
Evolutional model of the study area after 49 Ma. (**a**) Geodynamic model at magnetic isochrons C22/C21 (49 Ma to 44 Ma). GBR is a major oceanic plateau probably derived from Oki-Daito mantle plume^15^, which developed GR and UP; (**b**) after 41 Ma, BR, GR, and UP started to separate. Amagmatic seafloor spreading episode triggered the LOFZ formation; (**c**) present day geodynamic model. GBR: Great Benham Rise; BR: Benham Rise; UP: Urdaneta Plateau; GR: Gagua Rise; LOFZ: Luzon–Okinawa Fracture Zone.

Our results suggest that the BR, GR, and UP originated from a first proposed oceanic plateau named Great Benham Rise (GBR) between 49 and 41 Ma (Fig. 7). After 41 Ma, the magma propagated southeastward to the east of the LOFZ, developing a N–S trending amagmatic spreading episode, which also triggered the LOFZ movement. Thus, the BR, GR, and UP separated after 41 Ma. Nevertheless, since the GBR magma was supplied only to the east of the LOFZ in the WPB, the N–S trending second spreading episode only occurred in the east of the LOFZ.

## Conclusions

The West Philippine Basin (WPB) started spreading from magnetic isochron C26 and ceased spreading at C13. This spreading episode can be divided into magmatic and amagmatic episodes via magnetic age identification. Our results shows that the Gagua Rise (GR) occurred in between magnetic isochron C22 and C20, generating a V-shaped seamount chain and an overlapping spreading center system. The seismic reflection signal of the Moho reflection beneath the GR displays a discontinuous and medium amplitude, encountered frequently in other oceanic plateau areas. The crust accreted and thickened to ~ 12.7 km in GR, but it is still thinner than the BR (~ 15 km). The GR is also constrained by high residual free-air gravity anomalies, low Bouguer gravity anomalies and enhanced magnetic analytic signals. The magma propagation rate of the GR is estimated to be ~ 52.4 mm/yr to the west of the LOFZ, which is lower than ~ 94.4 mm/yr in the area to the east of the LOFZ. The GR occurrence age is estimated as ~ 49 Ma similar to that of BR (48 to 41 Ma), but the magmatic spreading episodes (53 to 43 Ma) happened to the east of the LOFZ. We first propose an oceanic plateau named Great Benham Rise (GBR) which includes GR, BR and Urdaneta Plateau (UP). We suggest that the GR was separated from the GBR at ~ 41 Ma by the right-lateral LOFZ motion. After 41 Ma, relict GBR (BR) only provided rest magma source to the east of the LOFZ. That is why there are no N–S trending amagmatic spreading episode to the west of the LOFZ.

## Supplementary Information

Supplementary Information 1.
