# Supplementary material for: Separation of Gagua Rise from Great Benham Rise in the West Philippine Basin during the Middle Eocene
Source: Sci Rep. 2021 Nov 5;11:21775. doi: 10.1038/s41598-021-01330-2 (PMC8571341; doi:10.1038/s41598-021-01330-2)
Supplement: Supplementary file 1 — Supplementary Information 1. [file 41598_2021_1330_MOESM1_ESM.docx]

**Supplementary Materials**

To better understand the magmatic distribution and crustal accretion of the Gagua mantle plume to the west of the Luzon-Okinawa Fracture Zone, the residual gravity anomaly was derived by subtracting the satellite-derived free-air gravity anomaly from the long wavelet gravity anomaly shown in Supplementary Figs. 1 and 2. The seismic data acquisition parameters are listed in Supplementary table one.


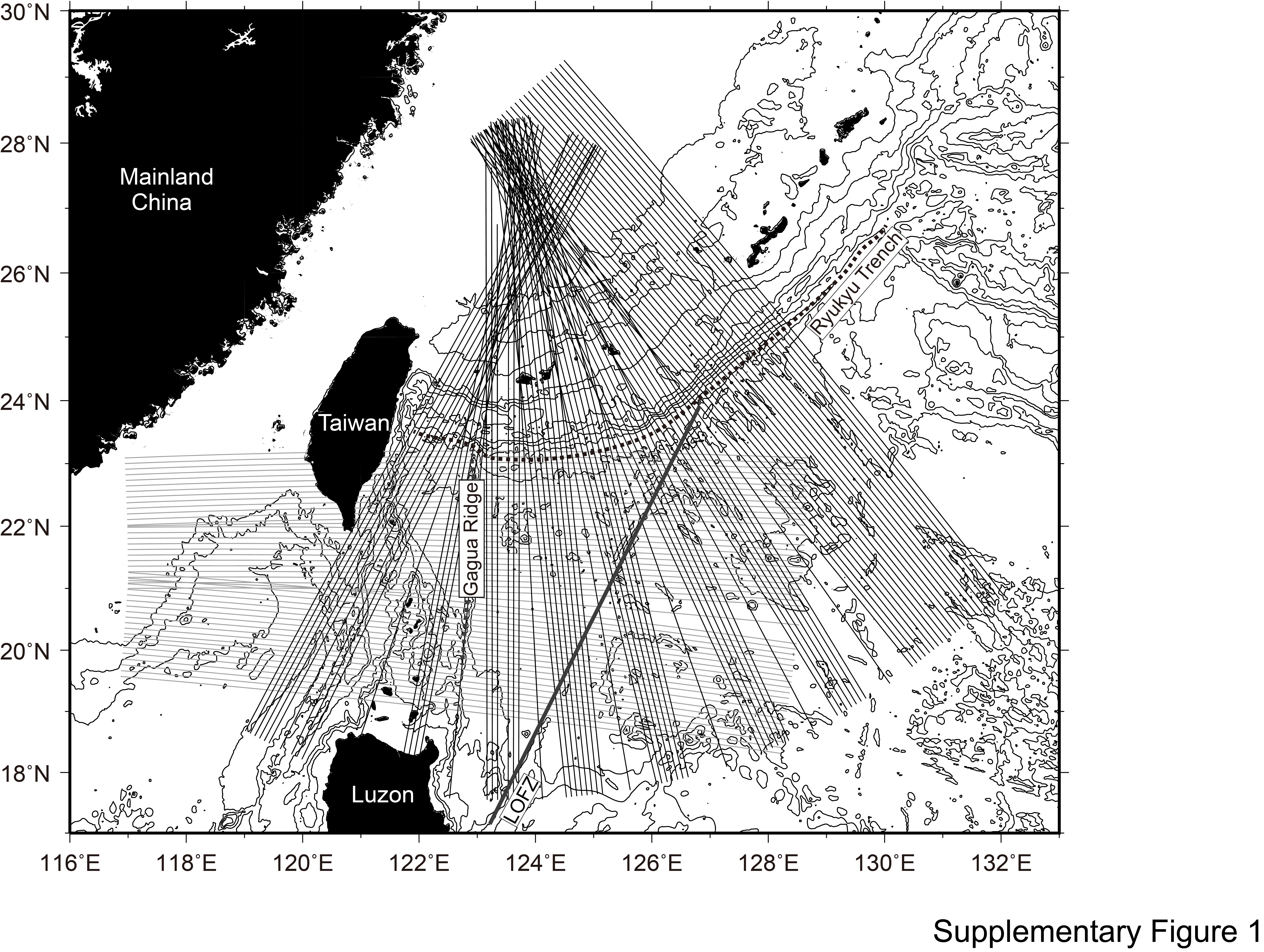


**Supplementary Fig. 1** Figure illustrates the profiles and area for long-wavelength gravity anomaly removal by applying a free-air gravity anomaly^18^ profile ensemble stack across the southern RT and Gagua Ridge. Black lines show the RT area and gray lines represent the Gagua Ridge area. The total profile distance is 1200 km, and the profile interval is 10 km.


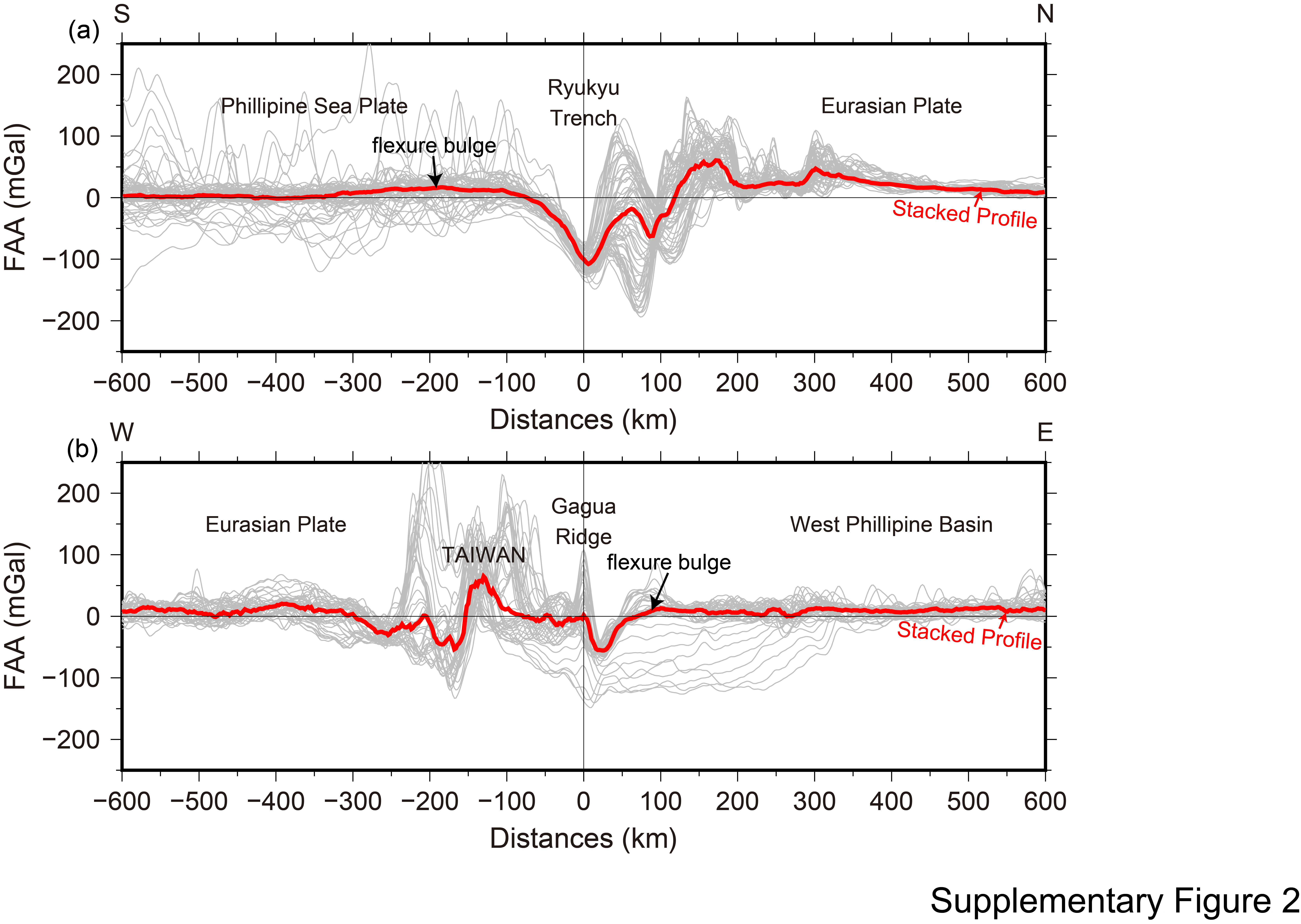


**Supplementary Fig. 2** 1 arc min × 1 arc min grid spaced free-air gravity anomaly profiles^18^ and ensemble stack profiles. (a) Free-air gravity anomaly profiles and their stacked profile across southern RT; (b) Free-air gravity anomaly profiles and their stacked profile across Gagua Ridge.

**Supplementary Table 1**: Seismic data acquisition parameters

| **Line Name** | **Line 01 (MGL0906-24)** | **Line 02 (MGL0908-10)** |
| --- | --- | --- |
| Seismic Source Total Volume (cubic inches) | 6600 | 6600 |
| Source Depth (m) | 8 | 8 |
| Total receiver channels | 468 | 468 |
| Group Interval (m) | 12.5 | 12.5 |
| Streamer Depth (m) | 9 | 9 |
| Recording Length (s) | 16 | 16 |
| Sampling Rate (ms) | 2 | 2 |
